# DAANISH: A mnemonic to aid in memorizing and recalling important strategies contributing to the prevention of perioperative stroke

**DOI:** 10.5339/qmj.2024.48

**Published:** 2024-09-16

**Authors:** Witoon Mitarnun

**Affiliations:** 1 Neurology Unit, Department of Internal Medicine, Buriram Hospital, Buriram, Thailand *Email: miwitoon@gmail.com

**Keywords:** Prevention, perioperative stroke, DAANISH, risk factors, mnemonic

## Abstract

**Background:**

Perioperative stroke, defined as a stroke occurring within 30 days post-surgery, typically emerges within the first week. The incidence of perioperative stroke among adults undergoing non-cardiac and non-neurological surgeries ranges from 0.1% to 1% after a surgical intervention. Mortality rates following perioperative stroke surge, reaching up to eight times higher than controls, with approximately one in four cases resulting in death. Fortunately, various strategies are available to potentially prevent perioperative strokes. However, the vast amount of data poses challenges for physicians in memorization and recall for clinical use. This study aims to summarize essential perioperative stroke prevention strategies and determine an effective mnemonic for their memorization and recall.

**Method:**

The initial search in PubMed focused solely on review articles published within the last 10 years. It utilized the keywords “perioperative stroke” and “prevention.”

**Results:**

An initial search found 39 articles, with two suitable for review. Using data from selected review articles, further searches were conducted on Google Scholar and PubMed for articles from 2000 to 2024, identifying 30 additional suitable references. From these 32 articles, the author developed a mnemonic, “DAANISH,” to aid in remembering strategies for preventing perioperative stroke.

**Conclusion:**

Implementing this mnemonic may help reduce the risk of perioperative stroke and improve patient outcomes. Future research is needed to confirm its effectiveness.

## 1. Introduction

Perioperative stroke, involving both ischemic and hemorrhagic cases, is defined as a stroke occurring within 30 days after surgery.^1^ Most perioperative strokes occur within the first week after surgery, with ischemic strokes representing over 95% of cases.^2,3^ According to previous studies, the frequency of perioperative stroke in patients undergoing non-cardiac, non-neurological surgery is estimated to be between 0.1% and 1%.^4,5^ The NeuroVISION trial demonstrated that among patients aged 65 years and older who underwent elective non-cardiac surgery, there was a 7% occurrence of silent perioperative stroke. This occurrence was linked to subsequent postoperative cognitive decline.^6^

The pathogenesis of perioperative ischemic stroke includes factors such as recent discontinuation of antithrombotic medications, previous significant cerebrovascular stenosis, anemia, intraoperative hypoperfusion, thromboembolism, fat embolism, endothelial dysfunction, new-onset postoperative arrhythmia, and enhanced coagulability due to surgery.^1,5^ On the other hand, the pathogenesis of perioperative hemorrhagic stroke involves uncontrolled hypertension, hyperperfusion resulting from carotid artery revascularization, and the use of antithrombotic medications.^1,5^

The mortality rate within 30 days is significantly higher for patients experiencing a perioperative stroke, reaching levels up to eight times greater than in control groups, with absolute mortality rates recorded at 22.3%.^5,7^ Fortunately, various strategies are available to potentially prevent perioperative strokes. However, the vast amount of data challenges physicians to memorize and recall for clinical use.^1,5^ To memorize extensive data, using mnemonics is an interesting method. Mnemonics offer valuable benefits in clinical practice by aiding in memorizing complex medical information and facilitating quick recall of essential data.^8^ Additionally, they minimize the risk of errors by reducing the likelihood of forgetting critical information.^8^ O’Hara et al. showed that mnemonic training yields long-term benefits for data recall.^9^ This study aims to highlight essential perioperative stroke prevention strategies and establish a mnemonic for easier memorization and recall.

## 2. Data Sources for Daanish Mnemonic

The initial search was conducted in PubMed, focusing on review articles published within the last 10 years, using the keywords “perioperative stroke” and “prevention.” This yielded 39 articles, of which two were found to be suitable for review (accessible, covering all areas of perioperative stroke prevention, and based on scientific evidence). Utilizing data from selected review articles, searches were then conducted on Google Scholar and PubMed for articles published between 2000 and 2024, resulting in the identification of 30 additional suitable references, as indicated in the reference section. From these 32 articles, the author devised a mnemonic named DAANISH (which means “knowledge,” “science,” and “wisdom” in Arabic^10^) to facilitate the memorization and recall of perioperative stroke prevention strategies, as outlined in Table 1. These strategies include:

### 2.1. D for delaying elective surgery post-stroke

Patients with a history of stroke face an increased risk of perioperative stroke, and this risk is influenced by the timing of the surgery in relation to their most recent stroke event.^11^ According to Jørgensen et al.’s study, stroke patients undergoing elective noncardiac surgery within 3 months of a prior stroke had the highest perioperative stroke risk, with an event rate of 11.95%, while those undergoing surgery 3–6 months and 6–12 months after the stroke had event rates of perioperative stroke of 4.48% and 1.78%, respectively.^11^

The explanation may be that cerebral autoregulation has been shown to be impaired following a stroke, especially during the first 3 months after its occurrence, particularly when the stroke is moderate to severe.^12^ Therefore, to minimize the risk of perioperative stroke, it is recommended to delay elective surgery for at least 6 months following a stroke if possible.^1,5^ The timing should be tailored to each individual, considering factors such as the risk of recurrent stroke, cardiovascular events, and the potential risks associated with delaying the surgery.

In the context of coronary artery disease, it is recommended that elective surgeries be deferred for a minimum of 14 days following angioplasty, 30 days following bare metal stent placement, and 1 year following drug-eluting stent placement.^1^

### 2.2. A for assessing the risk

The risk factors for perioperative stroke include surgical factors and patient factors. Smilowitz et al. found that surgeries increasing the risk of perioperative stroke, compared to general surgery, include vascular, neurological, thoracic, transplant, endocrine, skin/burn, and otolaryngology surgeries, with adjusted odds ratios of 11.97, 4.91, 3.78, 2.10, 1.70, 1.56, and 1.50, respectively.^13^ In terms of patient factors, at least 13 risk factors were identified in the previous study that increase the risk of perioperative stroke. These factors can be easily memorized as CHADS-ABCDEFGH, as shown in Table 2.^4,14–20^ The majority of these patient factors, with the exception of age, sex, and genetics, could be modified or controlled through medication or lifestyle adjustments before undergoing elective surgery.

Data on patient factors and surgical factors can be utilized to calculate scoring systems such as the CHADS_2_ score, Woo perioperative risk, and STRAS scoring system. These scores can then be employed for risk stratification for perioperative stroke. Data from 540,717 patients who underwent non-cardiac surgery showed that the CHADS_2_ score, an acronym for congestive heart failure, hypertension, age over 75 years, diabetes mellitus, and prior stroke or transient ischemic attack (TIA), is a useful and reliable tool for predicting perioperative stroke (Wilcox et al.)^7^ It is also noted for its user-friendly nature. Patients without perioperative stroke have a median CHADS_2_ score of 1 with an interquartile range of 0–2, while patients with perioperative stroke have a median CHADS_2_ score of 2 with an interquartile range of 1–3.^7^ Additionally, each increase in the CHADS_2_ score is associated with an odds ratio of 1.96 for an increased risk of perioperative stroke.^7^ However, a disadvantage of using the CHADS_2_ score is that it does not include surgical factors when considering the risk.

The scoring systems incorporating both patient and surgical factors, such as Woo perioperative risk and STRAS scoring systems, demonstrate high efficacy in perioperative stroke risk stratification.^19,20^ The Woo perioperative risk score was developed and validated in over 1 million patients (N = 1,165,750), incorporating nine parameters: age, history of coronary artery disease, history of stroke, emergency surgery, serum sodium, serum creatinine, hematocrit levels, American Society of Anesthesiologists (ASA) physical status class, and surgery type. It demonstrated high predictive accuracy for perioperative stroke, with an area under the curve (AUC) of 0.876.^19^ (The AUC summarizes a binary model’s performance, with higher values indicating better performance. Specifically, in this setting, it refers to the predictive power for perioperative stroke.) Furthermore, this score can assess not only the risk of perioperative stroke but also cardiac and mortality risks. It is accessible on the website (https://qxmd.com/calculate/calculator_823/woo-perioperative-risk).

In 2021, Platzbecker et al.^20^ introduced a scoring system named the Stroke after Surgery (STRAS). This system encompasses various factors such as age, sex, race/ethnicity, ASA physical status, history of ischemic stroke or TIA, carotid artery stenosis, patent foramen ovale, migraine, arterial hypertension, atrial fibrillation, emergency surgery, neurosurgery, and vascular surgery. The study revealed the effectiveness of STRAS screening in reliably identifying patients with an elevated risk of ischemic stroke within the first year post-surgery. The AUC was recorded at 0.88 in the validation cohort, surpassing other prediction instruments.^20^

The author suggests that all patients undergoing surgery should initially be evaluated with the CHADS_2_ score. Patients with a CHADS_2_ score of 2 or greater, or those undergoing a high-risk perioperative stroke operation, should be further evaluated using a more detailed scoring system, such as the Woo perioperative risk or STRAS scoring system.^19,20^

### 2.3. A for antithrombotic management

#### 2.3.1. When to stop and start antiplatelet

Aspirin, an irreversible cyclooxygenase inhibitor, should be discontinued 7 days before high-risk bleeding surgery.^1^ Cilostazol, a phosphodiesterase inhibitor, should be stopped 2 days prior to the procedure.^21^ Clopidogrel, prasugrel, and ticagrelor, all acting as adenosine diphosphate (ADP) receptor antagonists, need to be paused 5–7 days before surgery.^1^ Additionally, ticlopidine, also functioning as an ADP receptor antagonist, should be stopped 10–14 days before the anticipated surgery.^21^ All antiplatelet medications should be resumed when the bleeding risk has diminished.^5^ However, in patients with recent percutaneous coronary intervention or recent stroke, it is advisable to consult with a cardiologist or neurologist before discontinuing or initiating antiplatelet therapy.

#### 2.3.2. When to stop and start warfarin

Warfarin should be discontinued 5 days before surgery.^1,5^ If the international normalized ratio is below the target therapeutic range, it is advised to administer low-molecular-weight heparin (LMWH) as a bridge therapy for patients at high thromboembolic risk and some at intermediate risk (as determined by clinical judgment), discontinuing LMWH 24 hours before the operation.

Bridging is not recommended for patients at low risk of thromboembolism. Individuals in the low-risk category for perioperative thromboembolism include patients who have been prescribed warfarin for the following indications:^5^

- Mechanical valve indication with a bileaflet aortic valve prosthesis, without atrial fibrillation and without other risk factors for stroke.
- Atrial fibrillation and a CHADS_2_ score of 0–2 (assuming no prior stroke or TIA).
- Venous thromboembolism more than 12 months ago and without any additional risk factors.

Resume warfarin administration 12–24 hours post-operation and initiate LMWH administration 48–72 hours post-operation if there is no risk of bleeding.^1^

#### 2.3.3. When to stop and start direct oral anticoagulants (DOACS)

At present, there is no indication of heparin for bridging DOACs.

*Dabigatran perioperative management*:^1,5^

- For individuals with normal renal function: stop 2 days before a high-risk bleeding operation and 1 day before a low-risk one.
- For those with impaired renal function: stop 4 days before a high-risk bleeding operation and 2 days before a low-risk one.

Resume intake 2–3 days after a high-risk bleeding operation and 1 day after a low-risk one.

*Rivaroxaban, apixaban, and edoxaban perioperative management*:^1,5^

- Hold the medication for 2 days before high-risk bleeding operations and 1 day before low-risk ones.
- Resumption of medication takes place 2–3 days after high-risk bleeding operations and 1 day after low-risk ones.

### 2.4. N for normalized intraoperative parameters

Intraoperative management includes blood pressure management, ventilator setting strategies, and monitoring of end-tidal carbon dioxide. During surgery, blood pressure often drops, with mean arterial pressures falling below 20% of the baseline in up to 90% of cases.^22^ The explanation of intraoperative hypotension might be explained by anesthesia-related factors, blood loss, inadequate fluid replacement, medications, and pre-existing medical conditions. The consensus statement concluded that maintaining systolic blood pressures below 100 mmHg and mean arterial pressures below the range of 60–70 mmHg might be correlated with myocardial and kidney injuries.^23^ Additionally, a study by Somnuke et al. revealed that intraoperative hypotension, defined as an absolute mean arterial pressure <65 mmHg or a reduction of mean arterial pressure or systolic blood pressure of ≥ 20% from baseline, and the use of inotropes/vasopressors are linked to a 24-hour perioperative stroke, showing an adjusted odds ratio of 2.80 (95% CI: 1.08–7.24) for the risk of perioperative stroke.^16^ To date, due to insufficient evidence, specific blood pressure targets for preventing perioperative strokes remain undetermined. According to cerebral autoregulation mechanisms, a mean arterial pressure below 70 mmHg may diminish cerebral blood flow, increasing the risk of low-flow strokes, especially in patients with significant cerebrovascular stenosis. However, hypertension, defined as a mean arterial pressure of >100 mmHg, should be averted to lower the risk of surgical site bleeding, hemorrhagic stroke, myocardial infarction, cerebral swelling, and other end-organ damages.^24^ Therefore, physicians should consider maintaining a mean arterial pressure of 70–100 mmHg or keeping blood pressure within 20% of baseline, especially in patients at moderate or high risk for perioperative stroke.

For ventilation strategies, there is no data to support the ventilator strategies approach for the reduction of perioperative stroke. It is reasonable to consider a lung-protective ventilation strategy as part of an overall strategy to reduce postoperative complications.^5^ In terms of carbon dioxide levels, hypocarbia, which leads to cerebrospinal fluid alkalosis and subsequently triggers cerebral vasospasm, resulting in reduced cerebral blood flow,^25^ should generally be prevented in high-risk perioperative stroke patients. However, data from a retrospective case-control study led by Vlisides et al.^26^ revealed associations not only between intraoperative hypocarbia but also hypercarbia and an increased risk of stroke within 30 days after surgery. Specifically, end-tidal carbon dioxide levels of ≤30 or ≥45 mmHg were associated with a higher likelihood of stroke occurrence. Therefore, it is reasonable to maintain normocarbia to prevent perioperative stroke.

### 2.5. I for intraoperative neuromonitoring

Intraoperative neuromonitoring includes near-infrared spectroscopy, fiber optic jugular venous bulb oximetry, transcranial Doppler ultrasound, electroencephalography (EEG), and somatosensory evoked potentials (SSEP).^1,27–29^ The utilization of multimodal intraoperative neuromonitoring approaches enhances diagnostic accuracy compared to single methods, as demonstrated in the study by Thirumala et al.^29^, which found that simultaneous EEG and SSEP monitoring improves the likelihood of detecting periprocedural strokes after carotid endarterectomy. Moreover, detecting abnormalities during neuromonitoring can raise suspicion of a cerebrovascular event, thereby aiding in prompt stroke management.^30^ Nonetheless, there remains a lack of sufficient robust clinical evidence to conclusively confirm the beneficial impact of neuromonitoring.

### 2.6. S for symptomatic extracranial carotid artery stenosis revascularization

Significant extracranial carotid artery stenosis could impede blood flow to the corresponding side of the brain and potentially contribute to cerebral infarction in patients undergoing surgery. Udesh et al. analyzed 79,583 patients undergoing mitral valve surgery from 1999 to 2011 and discovered that carotid stenosis significantly increases the risk of perioperative strokes.^18^ The current guidelines recommend that individuals with severe extracranial carotid artery stenosis (>70%) and recent symptoms of ischemic stroke or TIA on the same side within the last 6 months (symptomatic) should seriously consider undergoing revascularization procedures such as carotid endarterectomy or carotid artery stenting.^31^ Additionally, symptomatic patients with moderate stenosis (50–69%) should also be considered for revascularization, as long as the surgical risk remains below 6%.^31^ However, it is generally unnecessary to conduct further evaluation of the carotid arteries in a patient with no history of prior stroke, TIA, or asymptomatic carotid bruit before surgery. The presence of a carotid bruit alone does not reliably indicate the severity of underlying carotid stenosis and has not been shown to increase the risk of stroke around the time of surgery.^32^

### 2.7. H for hemoglobin level targeting

Woo et al. discovered that anemic patients (hematocrit level less than 27%) faced a higher risk of perioperative stroke, with an odds ratio (95% CI) of 1.29 (1.08–1.55).^19^ This may be explained by anemia reducing the blood’s oxygen-carrying capacity due to decreased red blood cells or hemoglobin concentration. Consequently, vital organs, including the brain, may receive insufficient oxygen, especially during surgical stress. Blood transfusions offer crucial benefits, such as increased cerebral oxygen delivery, improved circulation, and treatment of anemia due to blood loss. However, they also entail risks, including an increased risk of thromboembolic events, transfusion reactions, transmission of infections, and acute lung injury, potentially leading to increased mortality. According to recommendation,^5^ physicians may consider a hemoglobin transfusion threshold of 8 g/dL for patients with recent stroke or significant cerebrovascular disease. Additionally, they may consider a transfusion threshold of 8–9 g/dL in patients with an acute perioperative stroke or ongoing bleeding and those experiencing hemodynamic instability.

## 3. Discussion

To the best of my knowledge, this review is the first to employ a mnemonic to assist in memorizing and recalling strategies for preventing perioperative stroke. The word “DAANISH” consists of seven letters and two syllables, with a positive and meaningful connotation that facilitates its memorization. Although the effectiveness of using DAANISH as a mnemonic to prevent perioperative stroke has not been studied, it has already been introduced to anesthesiologists and received a positive response in terms of ease of remembrance and coverage of all important issues for perioperative stroke prevention. Moreover, the mnemonic DAANISH, when utilized in the pre-anesthesia clinic, streamlined the management of high-risk perioperative stroke patients by serving as a checklist. This helped prevent the omission of essential information and enabled the rapid recall of key strategies for preventing perioperative strokes.

Future studies should investigate the effectiveness of the mnemonic DAANISH in both the short- and long-term. In the short term, the research could evaluate the ease of memorization and recall of strategies for perioperative stroke prevention. For long-term outcomes, a cohort study could assess the impact of this mnemonic on reducing the incidence of perioperative stroke.

## 4. Conclusion

The author presented the innovative mnemonic “DAANISH” as a tool to help remember important strategies that contribute to preventing perioperative stroke. It is hoped that physicians will adopt these strategies to reduce the risk of perioperative stroke and enhance patient outcomes.

## Conflict of Interest Statement

No conflict of interest in this review.

## Acknowledgments

I would like to thank my wife, Wenika Mitarnun, and my son, Daanish Mitarnun, for their support and encouragement.

## Ethics Statement

As this manuscript is a review article, ethical approval is not required.

## Figures and Tables

**Table 1.** “DAANISH” as a mnemonic for preventing perioperative stroke.

D	Delaying elective-surgery post-stroke
A	Assessing the risk
A	Antithrombotic management
N	Normalizing intraoperative parameters
I	Intraoperative neuromonitoring
S	Symptomatic extracranial carotid artery stenosis revascularization
H	Hemoglobin level targeting

**Table 2.** “CHADS-ABCDEFGH” serves as a mnemonic for patient factors related to perioperative stroke.

**Letter**	**Patient Factors**
C	Cardiovascular disease (AF, CHF, VHD, MI)
H	Hypertension
A	Age ≥ 65 years
D	Diabetic mellitus
S	Stroke or TIA history
A	ASA physical status
B	Beta-blocker (non-cardio selective) initiated during perioperative
C	COPD or current smoker
D	Dialysis or acute renal failure
E	Extracranial carotid artery stenosis
F	Female
G	Genetic (race/ethnicity)
H	Hematocrit levels
